# The Physiological Period Length of the Human Circadian Clock *In Vivo* Is Directly Proportional to Period in Human Fibroblasts

**DOI:** 10.1371/journal.pone.0013376

**Published:** 2010-10-15

**Authors:** Lucia Pagani, Ekaterina A. Semenova, Ermanno Moriggi, Victoria L. Revell, Lisa M. Hack, Steven W. Lockley, Josephine Arendt, Debra J. Skene, Fides Meier, Jan Izakovic, Anna Wirz-Justice, Christian Cajochen, Oksana J. Sergeeva, Sergei V. Cheresiz, Konstantin V. Danilenko, Anne Eckert, Steven A. Brown

**Affiliations:** 1 Neurobiology Laboratory for Brain Aging and Mental Health, Psychiatric University Clinics Basel, Basel, Switzerland; 2 Institute of Internal Medicine of the Siberian Branch of the Russian Academy of Medical Sciences, Novosibirsk, Russia; 3 Department of Pharmacology and Toxicology, University of Zurich, Zurich, Switzerland; 4 Centre for Chronobiology, Faculty of Health and Medical Sciences, University of Surrey, Guildford, United Kingdom; 5 Circadian Physiology Program, Division of Sleep Medicine, Brigham and Women's Hospital, Harvard Medical School, Boston, Massachusetts, United States of America; 6 Department of Dermatology, University Hospital Basel, Basel, Switzerland; 7 Centre for Chronobiology, Psychiatric University Clinics Basel, Basel, Switzerland; 8 Institute of Cytology and Genetics of the Siberian Branch of the Russian Academy of Sciences, Novosibirsk, Russia; University of Wuerzburg, Germany

## Abstract

**Background:**

Diurnal behavior in humans is governed by the period length of a circadian clock in the suprachiasmatic nuclei of the brain hypothalamus. Nevertheless, the cell-intrinsic mechanism of this clock is present in most cells of the body. We have shown previously that for individuals of extreme chronotype (“larks” and “owls”), clock properties measured in human fibroblasts correlated with extreme diurnal behavior.

**Methodology/Principal Findings:**

In this study, we have measured circadian period in human primary fibroblasts taken from normal individuals and, for the first time, compared it directly with physiological period measured *in vivo* in the same subjects. Human physiological period length was estimated via the secretion pattern of the hormone melatonin in two different groups of sighted subjects and one group of totally blind subjects, each using different methods. Fibroblast period length was measured via cyclical expression of a lentivirally delivered circadian reporter. Within each group, a positive linear correlation was observed between circadian period length in physiology and in fibroblast gene expression. Interestingly, although blind individuals showed on average the same fibroblast clock properties as sighted ones, their physiological periods were significantly longer.

**Conclusions/Significance:**

We conclude that the period of human circadian behaviour is mostly driven by cellular clock properties in normal individuals and can be approximated by measurement in peripheral cells such as fibroblasts. Based upon differences among sighted and blind subjects, we also speculate that period can be modified by prolonged unusual conditions such as the total light deprivation of blindness.

## Introduction

Nearly all aspects of human daily behavior and physiology are governed by a master clock in the suprachiasmatic nuclei (SCN) of the hypothalamus. This intrinsic oscillator not only governs sleep and wake timing, but also rhythms of temperature, the hormones melatonin and cortisol, mood and cognitive acuity, cardiac, respiratory, and renal function, and most aspects of digestion and detoxification [1]. These rhythms are entrained to 24 hours by the environmental light-dark cycle primarily via a subset of photosensitive retinal ganglion cells that project directly to the SCN [2]. Using multiple hormonal and neuronal signals, the SCN “master” clock in turn entrains peripheral clocks of similar molecular mechanism present in most cells of the body [3].

In humans and other organisms, the timing of 24-hour behavior is governed by the period length of the circadian oscillator. This period is approximately, but not exactly, 24 hours long (“*circa diem*”), and has a reported population range of 23.47–24.64 in laboratory conditions [4]–[11] Short periods lead to behavior occurring at an earlier clock time in some individuals (so-called “larks”), and long periods to later timing of behavior in others (“owls”) [9], [12]–[14], at least in young adults. Mutations in clock genes affect period length, and can lead to circadian rhythm sleep disorders such as Advanced Sleep Phase Syndrome (ASPS) [15]. Measurement of circadian period length is therefore a useful step in assessing circadian clock function, either experimentally or clinically.

Determination of the period of circadian behavior in sighted subjects requires prolonged subject observation under controlled-light laboratory conditions. In humans, these studies are expensive and labor-intensive. In one protocol, subjects are kept for multiple days under ‘constant routine’ conditions consisting of sustained wakefulness or scheduled sleep episodes under continuous dim light with constant posture and frequent isocaloric meals [16], [17]. Alternatively, circadian period can be measured under constant dim light for several weeks [18]. Finally, it can be measured under ‘forced desynchrony’ (FD) conditions where the sleep-wake cycle is scheduled to day-lengths that are outside the range of entrainment for the circadian pacemaker, conditions which force the clock to exhibit its endogenous period. Since it is not ethically possible to keep human subjects in constant routine protocols for prolonged periods, FD protocols are thought to offer greater precision. [4]. A further special case employs totally blind individuals, whose circadian pacemaker cannot be entrained by the environmental light-dark cycle. Although under certain circumstances these individuals can become entrained to the 24-hour day via nonphotic cues such as exercise, food, and activity [19], [20], in other cases they exhibit ‘non-24-hour’ rhythms in melatonin, cortisol or temperature under both laboratory and real-world conditions that provide a direct estimate of their physiological period [21].

Given the intensive experimental control required to assess human physiological period, our laboratories have taken advantage of the duplication of the molecular mechanism of the circadian clock in most mammalian tissues to measure period length in human fibroblasts. To do this, we infect cells with a lentiviral reporter containing the luciferase gene under control of a circadian promoter. Period length in infected cells can then be measured via real-time bioluminescence [22]. In mice with mutations in circadian clock genes, a qualitative correlation has been observed between behavioral period measured by wheel running activity and fibroblast period measured by lentiviral reporter; and in humans, extreme early-type individuals had on average shorter fibroblast periods than extreme late-types [13]. Although these correlations among mutant animals or extreme chronotypes are promising, it remains unclear how well human period length is predicted from measurements in peripheral tissues in ordinary individuals, and whether these values correlate with sleep-wake timing *in vivo*.

In this report, we have for the first time directly compared estimates of human physiological circadian period with the period measured in fibroblasts from the same subjects. We used three different subject groups whose physiological period was estimated via different protocols, and which showed different population averages for physiological period. In each trial, the correlations that we observed suggested that this easy cellular method could be used as a biomarker for human clock properties.

## Results

### Human fibroblast circadian period *in vitro* correlates with estimates of human physiological period measured *in vivo* in normal individuals

As described in Methods, we recruited subjects that had participated previously in one of five different studies of physiological period in three countries (Basel, Switzerland; Novosibirsk, Russia; and Guildford, United Kingdom). Four protocols estimated physiological period of sighted subjects under controlled laboratory conditions designed to minimize the effects of environmental light, exercise, food, and sleep upon the workings of the circadian oscillator. Physiological period was determined by the timing of melatonin, a hormone produced at night in circadian fashion and measurable in human saliva. A fifth study focused upon totally blind individuals. For such people, free-running physiological period has been shown previously to remain independent of the solar day even in a home environment under some circumstances [21]. Hence, subject period could be measured at home over several weeks via the periodicity of the melatonin metabolite 6-sulfatoxymelatonin excreted in urine.

**Figure 1:**
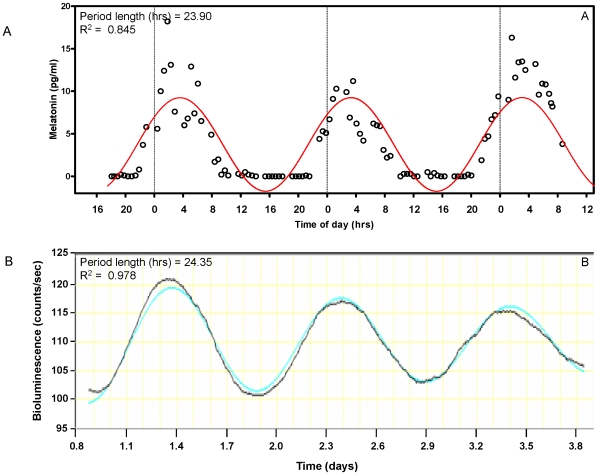
Determination of circadian period *in vivo* and *in vitro*. A. *In vivo* period length was obtained from salivary melatonin content profiles of normal sighted subjects under a constant routine protocol for three consecutive cycles. Data from one representative subject (Start25) are shown. Open circles, melatonin measurement values. B. From the same subject, fibroblast cultures were obtained from two separate biopsies and infected with a lentiviral bioluminescent circadian reporter. After synchronization with dexamethasone, circadian oscillations in bioluminescence were recorded from eight measures over five days. Three cycles of this oscillation are shown aligned with the physiological data of part A. Period calculations for both panels were conducted by cosinor fitting, and the best-fit curve is shown in color, along with its period length and goodness of fit R2.

From each subject, two skin biopsies were taken and fibroblasts were cultivated from them. The circadian period length of these fibroblasts was then measured via transduction of a lentiviral circadian reporter and subsequent long-term bioluminescent monitoring. Figure 1B shows a portion of data from fibroblasts of the same subject whose physiological period is shown in Figure 1A.

**Figure 2:**
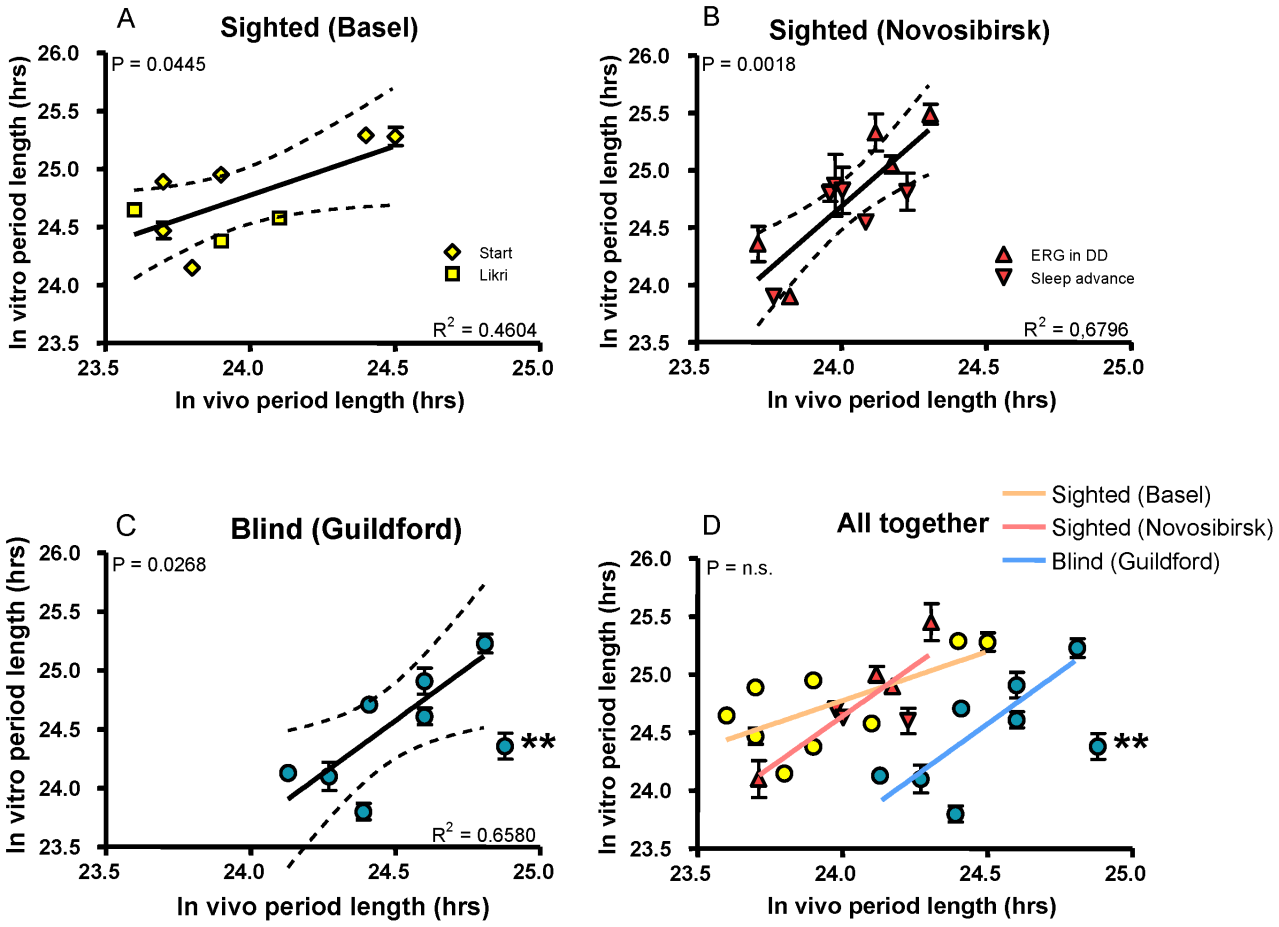
Relationship between physiological period length and fibroblast period length. A. *In vitro* period length was obtained from skin fibroblasts from nine sighted human subjects of normal chronotype, and compared to physiological period in the same individuals measured in two constant routine studies (Basel Start and Likri). The solid line shows the best-fit linear regression (1^st^ polynomial order) represented by the data, and the dashed lines represent 95% confidence intervals to the indicated regression line, with goodness-of-fit R2 shown at lower right. The overall p-value (vs. null-hypothesis slope of 0) is shown at upper-left. For this panel and also panels B and C, identical statistical measures are depicted. In addition, the range and average for each group of subjects are shown in Table 1, and data for individual subjects are listed in Methods S1. B. The same comparison was performed for 11 sighted subjects whose physiological period was measured in controlled laboratory conditions in two studies (Novosibirsk). C. A further comparison was performed for 8 totally blind subjects whose physiological period was measured at home (Guildford). Fibroblasts from the asterisked subject showed abnormal clock properties *in vitro* at different temperatures and were excluded from statistical analysis. D. The results of all five studies are graphed on the same axes: Yellow, Basel; Red, Novosibirsk; Blue, Guildford.

For all subjects, estimates of physiological *in vivo* period were compared to fibroblast period measured via transduction of a lentiviral circadian reporter and subsequent long-term bioluminescent monitoring. The results from 9 subjects measured from two studies in Basel are shown in Figure 2A. Results from a further 11 subjects participating in two studies in Novosibirsk are shown in Figure 2B. Finally, results from 8 totally blind individuals measured in Guildford are shown in Figure 2C. All data are graphed together in Figure 2D. In all groups, positive correlations were observed between fibroblast period and physiological period measured in the same subjects, and Bland-Altman statistics among all subjects show an absence of systematic error between measurements *in vitro* and *in vivo* (Figure S1). Table 1 shows the specific values and correlation coefficients obtained from each subject population.

**Table 1:** Period lengths obtained from subjects by physiological measurements and from fibroblast molecular analyses.

Subject location	N	τ *in vitro*	τ *in vitro*	r^2^
**Basel**	9	24.00±0.33	24.71±0.38	0.46
		(23.60–24.50)	(24.15–25.29)	
**Novosibirsk**	11	24.07±0.20	24.77±0.42	0.68
		(23.71–24.31)	(24.10–25.45)	
**Guildford**	8	24.52±0.27	24.46±0.48	0.66
		(24.13–24.92)	(23.80–25.23)	

### Blind individuals showed longer physiological period but equivalent fibroblast period

**Figure 3:**
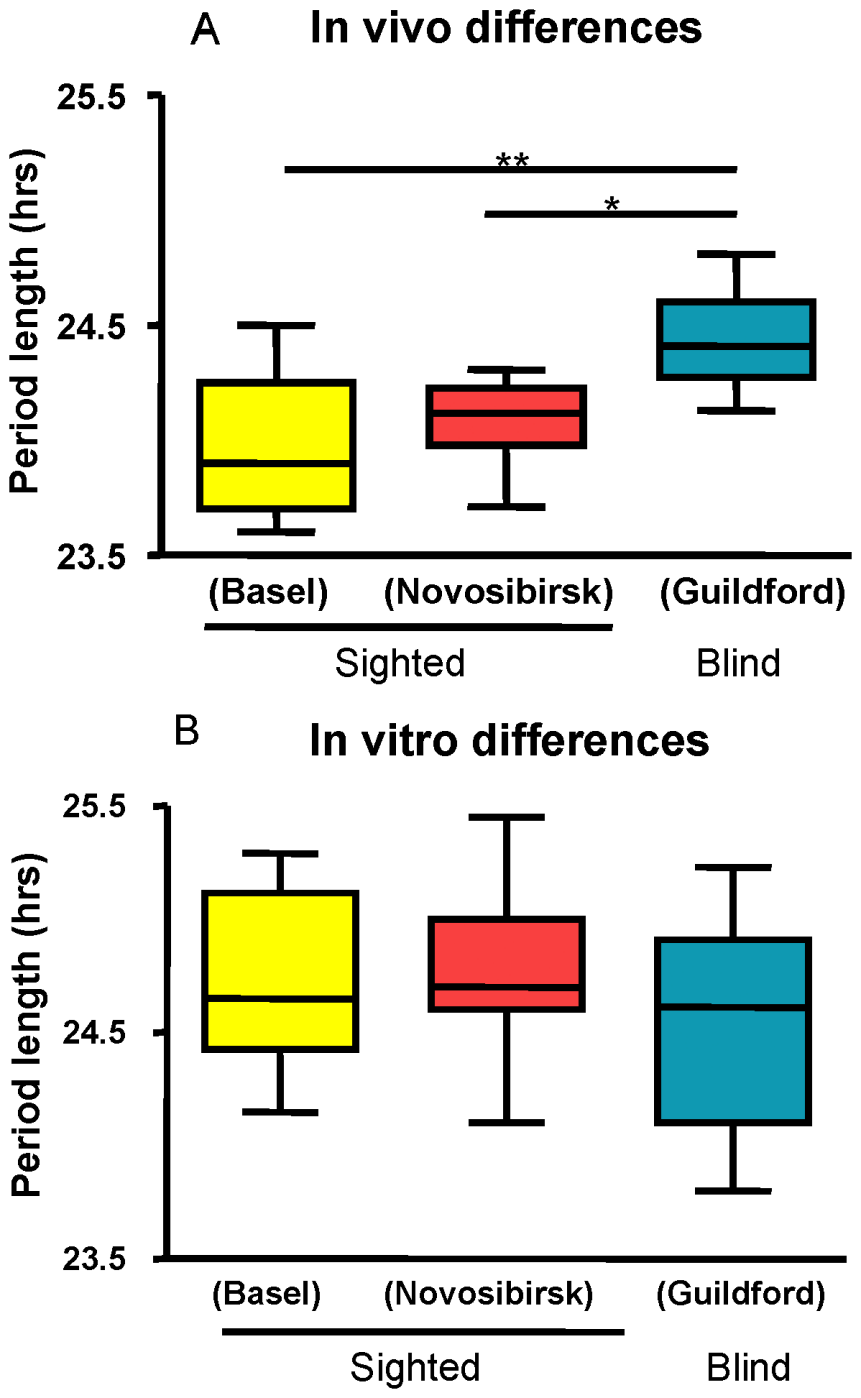
Relationship between population averages of human physiological period length and fibroblast period length. A. The average *in vivo* period lengths obtained from both protocols with sighted individuals were significantly different from that of blind subjects (p<0.01, 0.05). B. Nevertheless, the fibroblast-derived values were not significantly different among all three populations. (1-way Anova). Data are presented as a standard boxplot, with bars showing the smallest and largest observed values, and box dimensions and midline reflecting lower quartile (Q1), median (Q2), and upper quartile (Q3) values.

Average physiological period lengths were significantly longer for the blind subjects than for the sighted subjects (Figure 3A), as reported previously [4], [21]. Surprisingly, average fibroblast period was the same for these three populations when measured under identical conditions (Figure 3B). In other words, cellular fibroblast periods were similar among the groups of subjects even though physiological periods varied. Therefore, fibroblast measurements succeeded in capturing inter-individual differences in period among members of each group (whether they were blind or sighted), but were insensitive to apparent differences between blind and sighted individuals.

## Discussion

The fundamental mechanism of the mammalian circadian clock is cell-autonomous, and is based upon feedback loops of transcription and translation that use identical components in most cells of the body [23]. In fact, long-term recordings suggest that the intrinsic clocks in peripheral cells like fibroblasts are probably as robust as those in the “master clock” of the brain SCN [24]. Probably because of these mechanistic similarities, our laboratory has been able to show previously that clock properties of fibroblasts taken from subjects of extreme chronotype (i.e. “larks” and “owls”) generally correlate with the results of questionnaires about subject daily behavior. Many of these extreme subjects, however, showed normal fibroblast period length. We ascribed their behavior to alterations in other clock properties like amplitude [13].

Thus, it remains unclear to what degree the genetically encoded period length of the human circadian clock, as reflected in peripheral tissues like fibroblasts, controls daily behavior in normal individuals whose period lengths vary relatively little. Herein, we have rigorously examined this matter by comparing directly circadian period length measured from human fibroblasts and that estimated physiologically in the same individuals, under different experimental conditions. In the five studies from three laboratories that we have analyzed, we have observed good correlations between period lengths of the circadian oscillator *in vivo* and *in vitro*, even among individuals whose clock properties vary very little (Figure 2).

**Figure 4:**
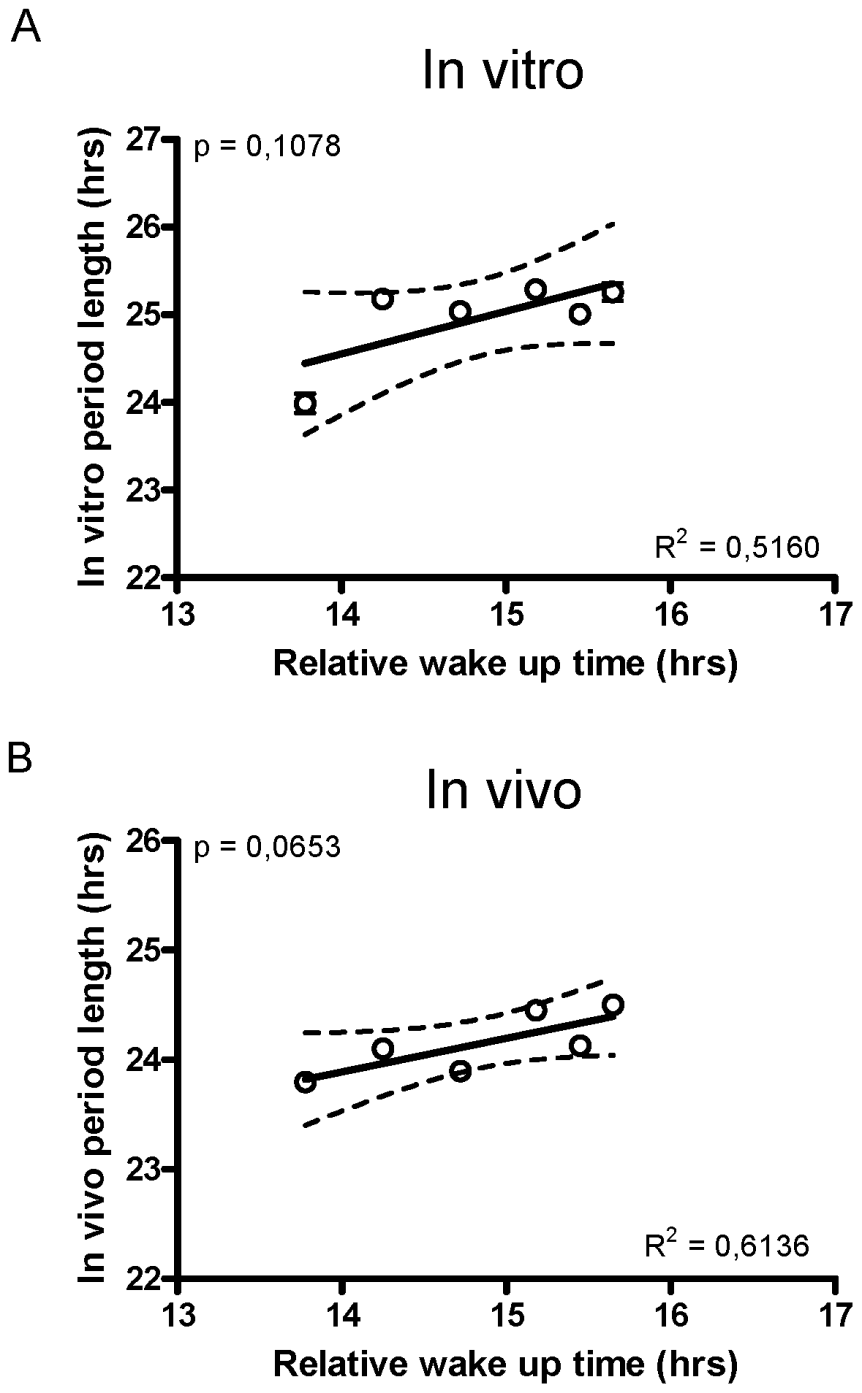
Circadian period and wake timing. A. Period length measured in fibroblasts is plotted against the circadian phase angle of sleep, calculated as the difference between the measured time of dim-light melatonin onset on the first day of the protocol and the time of subject spontaneous waking that morning. This value could only be determined in one of the five studies presented (Basel constant routine). Linear regression and statistical analyses were as in Figure 2. B. Period length *in vivo* is plotted versus the same sleep phase angle.

*In vivo*, circadian period length under free-running conditions has been shown previously to correlate well with circadian phase under entrained conditions. This circadian phase is best reflected in outputs of the circadian oscillator, such as sleep-wake timing [9], [14]. In retrospect, we were interested to know to what extent fibroblast period could predict sleep-wake timing, but our studies were not designed to test this hypothesis and in most cases the rigorous routines of our subjects also controlled sleep-wake. Nevertheless, for the six subjects participating in a constant routine protocol in Basel, physiological circadian phase was measured on the first night of in-patient study via dim-light melatonin onset, and spontaneous wakeup time the next day by EEG. By comparing these two times, we could estimate a “phase angle” of sleep timing with respect to melatonin, which from previous studies should correlate with period [14]. In our case, we could directly compare this phase angle with subject circadian period length measured both *in vitro* and *in vivo*. A trend was observed between sleep timing and circadian period length *in vivo* (Figure 4A) and in fibroblasts (Figure 4B), but limited subject number prevented it from reaching significance. A larger study would be needed to answer this question more rigorously.

Approximation of human circadian period by cellular assays could present important advantages. The measurement of human circadian period *in vivo*, either via constant routine or forced desynchrony protocols, is expensive and labor-intensive. An *in-vitro* measure of human circadian period, if validated *in vivo*, could provide an attractive lower-budget alternative. Nevertheless, it should be noted that fibroblast period measures are not a direct proxy for measures of physiological period: for some subjects, considerable differences between the two were observed, and physiological period differences between sighted and blind subjects were absent entirely in fibroblasts.

While it is too soon to be able to draw firm conclusions, it is possible that fibroblast period might prove more useful than physiological period in certain applications. For example, determination of period length in fibroblasts under carefully controlled conditions might permit separation of genetic effects upon basic clock mechanism from environmental influences present in more complex physiological measurements. Such influences are vividly illustrated by the measurement of sighted and blind subjects included in the present paper. In ours and other published studies of sighted subjects, various protocols have all given period values close to 24 hours [4], [6], whereas period length measured in blind subjects at home are longer, and average 24.5 hours [21] (See also Figure 3A,B). This discrepancy is unlikely to result from differences in methodology: recent data illustrates that period assessments in field studies of blind individuals are comparable to period assessment under forced desynchrony in the same subjects (Hull et al., *in press*). In reverse, two groups of sighted subjects in our study were measured in near total darkness (<0.2lux, Novosibirsk), and showed the same shorter period of the sighted subjects that we estimated with constant routine (Basel), or that others have estimated in forced-desynchrony protocols [4].

If the difference is not due to methodology, a basic question arises: are the shorter periods of sighted subjects “aftereffects” of entrainment to light, or is there a fundamental genetic difference between these groups? Our measurements suggest that it is unlikely that this difference is genetic: average fibroblast period was the same in all groups that we investigated, blind and sighted (Figure 3), but still reflected accurately genetic differences causing short and long physiological periods within each group. It is possible, of course, that fibroblast clocks are sensitive to some types of genetic variation but insensitive to others. Nevertheless, in our study we favor the hypothesis that circadian properties between blind and sighted groups differed for physiological reasons rather than genetic ones: for example, human physiological period length might be modified by prior light history and/or retinal function [25]. In support of this idea, individuals recently entrained to a simulated Martian day length showed a longer period length afterward [26].

It should be mentioned that the period of fibroblasts is not invariant, either: their circadian clocks are temperature-overcompensated [27], [28]. Thus, whereas normal biochemical reactions slow as temperature decreases, circadian clocks increase in speed. As a result, period length measured from fibroblast cells *in vitro* varies with the temperature at which the measurement is conducted. Not surprisingly, the best correlations with period length *in vivo* are seen at physiological temperatures. In addition, measurements conducted at different temperatures are correlated, preserving inter-subject differences (Figure S2A). Interesting exceptions exist, however. For example, one subject showed almost perfect temperature compensation at multiple temperatures, in stark contrast to the temperature overcompensation seen in all other subjects (Figure S2B). This subject also showed a significant difference between period *in vivo* and period *in vitro* (asterisked subject in Figure 2C). Although the meaning of this difference is uncertain, we suggest that inter-individual genetic variations also exist in the (thus far unknown) loci implicated in temperature compensation by the human circadian oscillator.

In the extreme case, our experiments suggest that a measurement of “true” circadian period emanating from the suprachiasmatic nucleus may be unrealistic. Both *in vivo* and *in vitro*, the observed period appears to be altered by environmental factors. Fibroblast period can be altered by temperature, and physiological period might be sensitive to prior light history and/or retinal function. Nevertheless, measurement of fibroblast period at precise physiological temperatures offers a reasonable approximation of physiological period determined by more rigorous measures. Both values, in spite of their variance, show strong inter-individual differences independent of environment or method, and will likely provide exciting clues to understand the genetic basis of human daily behavior and its influence upon health.

## Methods

### Ethical Permission

The study protocol, screening questionnaires, and consent forms were approved by the relevant ethical committee (the Ethical Committee of Basel, Switzerland; the Ethical committee of the Institute of Internal Medicine, SB RAMS, Novosibirsk, Russia; the University of Surrey Advisory Committee on Ethics; and the Moorfields Eye Hospital Ethics Committee) and conform to the Declaration of Helsinki. Informed consent was obtained from all subjects.

### Subject selection

Subjects for this study were individuals who have previously or concurrently participated in chronobiological studies of the authors in Basel, Novosibirsk, or Guildford, and were additionally recruited to donate skin biopsies for this study. In two studies (Basel, Novosibirsk), extreme chronotypes were specifically excluded. Where noted, physiological measurements have been reported previously in other contexts. In brief, study participants consisted of nine sighted subjects (6 men, 3 women, mean age ± S.D. 48.3±21.9 years) recruited in Basel, eleven sighted subjects (2 men, 9 women, mean age ± S.D. 25.5±7.8 years) recruited in Novosibirsk, and eight subjects with no perception of light (7 men, 1 woman, mean age ± S.D. 54.6±7.4 years) recruited in Guildford. Further information is cited in Methods S1.

### Circadian period determination *in vivo*

In order to ensure that the correlations presented herein were not dependent upon a particular method of determining circadian period, each subject group described above participated in a different protocol for the measurement of physiological period *in vivo*. Detailed descriptions of each protocol are listed in Methods S1. In brief, sighted subjects (Basel and Novosibirsk subject groups) were required to adhere to a regular sleep-wake schedule for 5–14 days prior to admission to the laboratory. Subsequently, subjects were maintained under various sets of “constant” conditions designed to eliminate environmental influences upon the circadian oscillator: a 60-hour multiple nap protocol or a 40-hour sleep deprivation protocol (Basel) under constant dim-light (<8 lux) conditions with constrained posture and frequent scheduled meals; or a 4 to 9-day protocol under near-total darkness (<0.1 or 0.2 lux) with normal scheduled sleep episodes (Novosibirsk). Saliva samples were collected at intervals of 0.3–3 hours and analysed for melatonin content through a direct double-antibody radioimmunoassay (Bühlmann Laboratories, Schönenbuch, Switzerland). Blind subjects (all with no perception of light, Guildford), were studied in their own homes for 3–5 consecutive weeks. No attempt was made to alter the lifestyles of the individuals during the study. For such people, free-running physiological period has been shown previously to remain independent of the solar day even in a home environment [21]. For 48 hours each week, subjects collected sequential ∼4-hourly urine samples during the day plus an ∼8-hour overnight sample. Urinary 6-sulphatoxymelatonin (aMT6s) concentrations were measured as described previously [29]. Period lengths were determined by the timing of intervals between melatonin rise, either by regression analysis in longer protocols (Novosibirsk, Guildford), or by cosinor analysis for shorter ones (Basel).

### Measurement of fibroblast circadian period length

Subjects from each of the studies presented above were recontacted to participate in the present investigation by donating skin biopsies. The time elapsed between measurements *in vivo* and fibroblast donation, as well as detailed protocols, are indicated in Methods S1. Briefly, two cylindrical 2-mm diameter cutaneous biopsies were taken from the buttocks or upper arms of each subject. Fibroblasts were isolated from biopsies and infected using *Bmal1*::luciferase lentivirus as described previously [22]. Five days or more after human fibroblast infection, circadian rhythms were synchronised by 100 nM dexamethasone (Sigma), and light output was measured (3 measurements per biopsy; total of 6 measurements per subject) in the presence of 0.1 nM luciferin in specially built light-tight atmosphere-controlled boxes for at least 5 days. Period determination was accomplished by cosinor analysis of data between the second and the fifth day of measurement. Values are presented as mean plus or minus standard error from four to eight measurements.

## Supporting Information

Figure S1 Bland-Altman statistics for period measurements. For each subject, average period length (*in vivo* and *in vitro*) is plotted against the difference between the two measurements, expressed in standard deviations from the mean.
(0.11 MB TIF)

Figure S2 Comparison of fibroblast period length measured at 36.5 degrees C and at 37.0 degrees C incubator temperature. A. Period length was measured from skin fibroblasts of blind and sighted subjects (from Guildford and Novosibirsk) at two different incubator temperatures, and plotted in comparison. Most subjects showed a similar augmentation in period at the higher temperature (1.1+/−0.3 hours). B. Extreme temperature compensation properties in one subject; this individual (S45) is marked with an asterisk in Figure 2. Fibroblast period lengths at 36.5, 37.0, and 37.5 degrees C are shown for this subject (left) versus another representative subject (S43) (right).
(0.17 MB TIF)

Methods S1 (0.16 MB DOC)
