# Supplementary figures and images for: The Physiological Period Length of the Human Circadian Clock In Vivo Is Directly Proportional to Period in Human Fibroblasts
Source: PLoS One. 2010 Oct 15;5(10):e13376. doi: 10.1371/journal.pone.0013376 (PMC2958564; doi:10.1371/journal.pone.0013376)

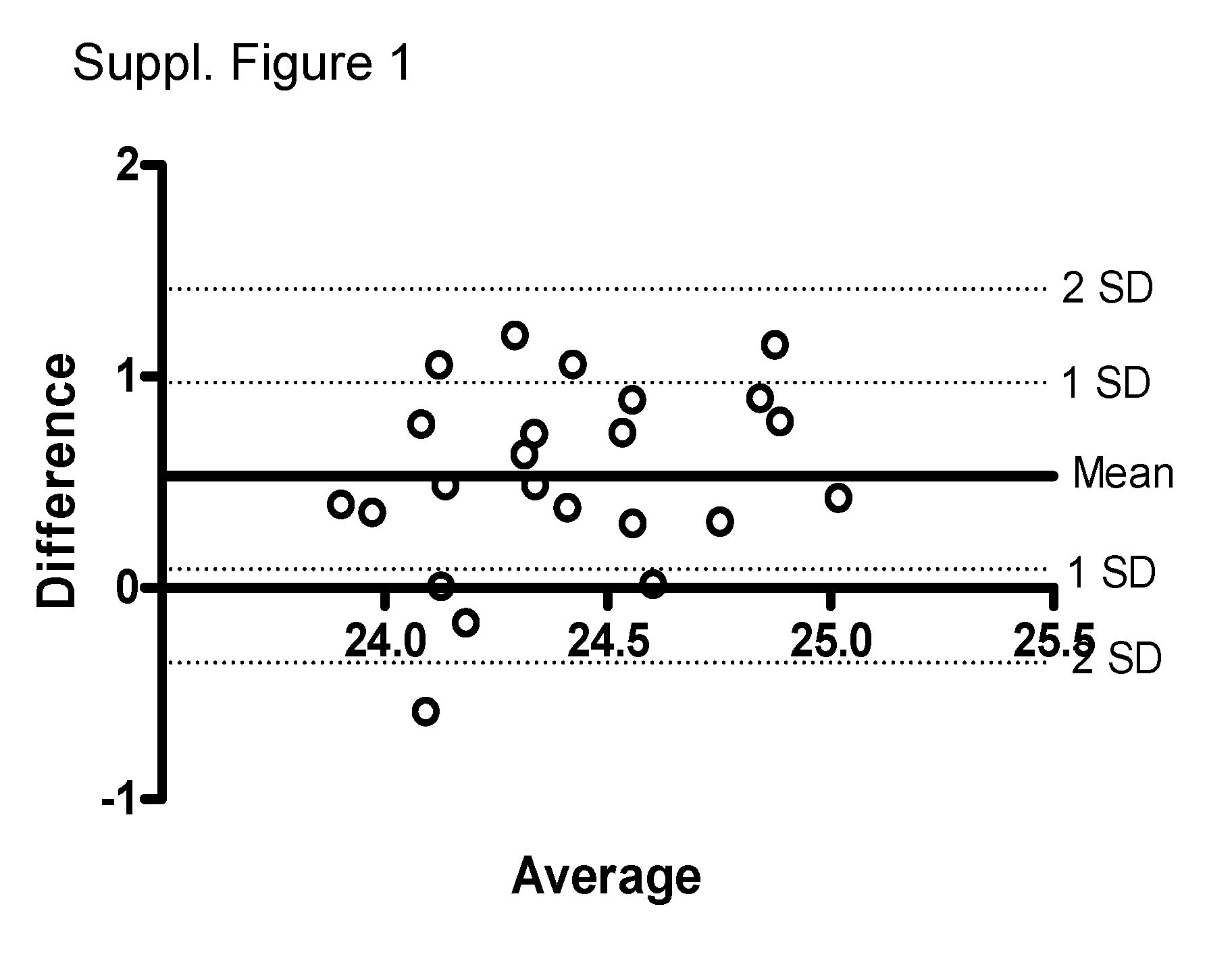

Supplement: Figure S1 — Bland-Altman statistics for period measurements. For each subject, average period length (in vivo and in vitro) is plotted against the difference between the two measurements, expressed in standard deviations from the mean. (0.11 MB TIF) [file pone.0013376.s001.tif]

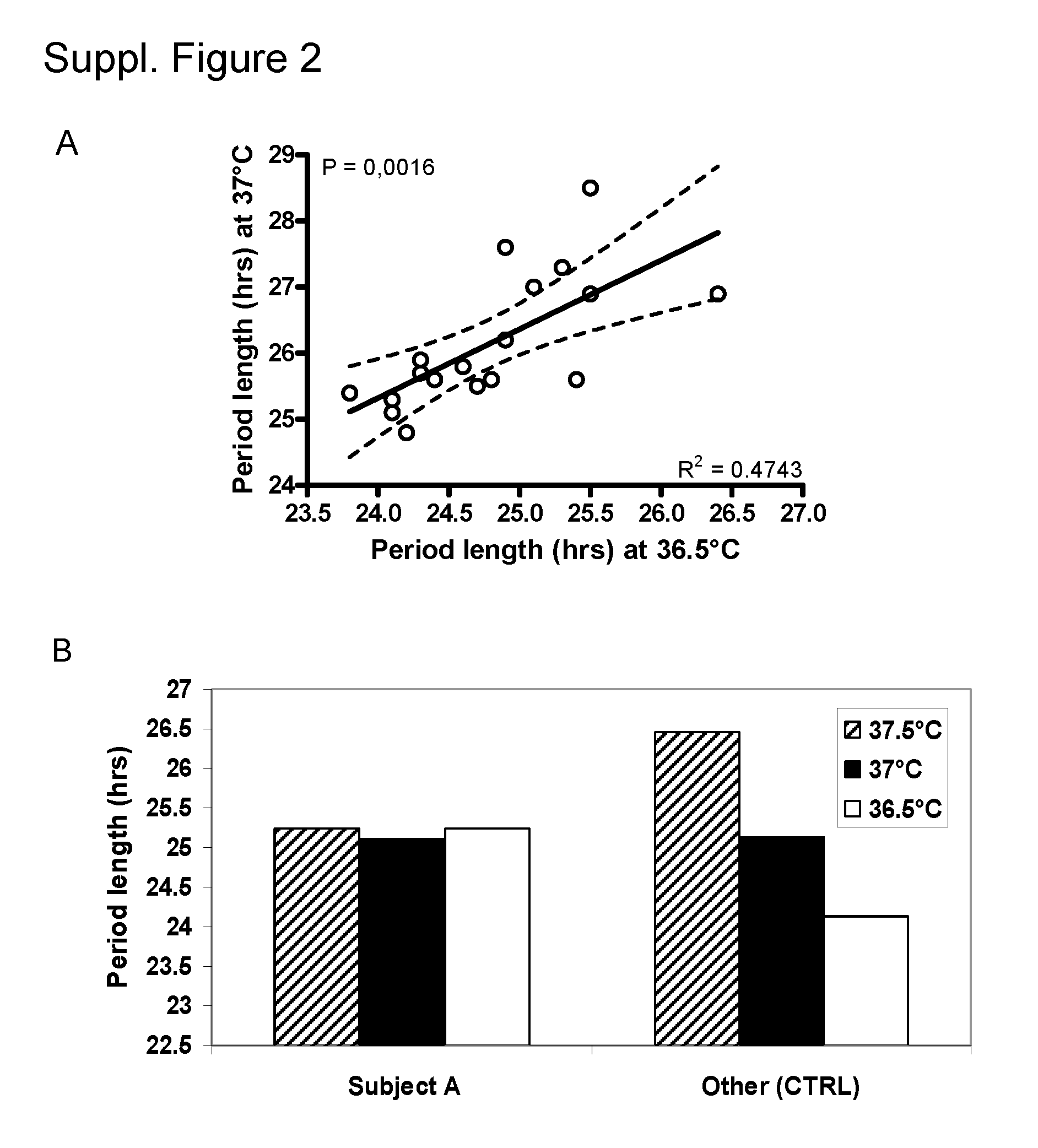

Supplement: Figure S2 — Comparison of fibroblast period length measured at 36.5 degrees C and at 37.0 degrees C incubator temperature. A. Period length was measured from skin fibroblasts of blind and sighted subjects (from Guildford and Novosibirsk) at two different incubator temperatures, and plotted in comparison. Most subjects showed a similar augmentation in period at the higher temperature (1.1+/−0.3 hours). B. Extreme temperature compensation properties in one subject; this individual (S45) is marked with an asterisk in Figure 2. Fibroblast period lengths at 36.5, 37.0, and 37.5 degrees C are shown for this subject (left) versus another representative subject (S43) (right). (0.17 MB TIF) [file pone.0013376.s002.tif]
