# Supplementary material for: The Physiological Period Length of the Human Circadian Clock In Vivo Is Directly Proportional to Period in Human Fibroblasts
Source: PLoS One. 2010 Oct 15;5(10):e13376. doi: 10.1371/journal.pone.0013376 (PMC2958564; doi:10.1371/journal.pone.0013376)
Supplement: Methods S1 — (0.16 MB DOC) [file pone.0013376.s003.doc]

**SUPPLEMENTARY METHODS**

**Subject selection and ethical permission.**

The study protocol, screening questionnaires, and consent forms were approved by the relevant ethical committee (the Ethical Committee of Basel, Switzerland; the Ethical committee of the Institute of Internal Medicine, SB RAMS, Novosibirsk, Russia; the University of Surrey Advisory Committee on Ethics; and the Moorfields Eye Hospital Ethics Committee) and conform to the Declaration of Helsinki. Informed consent was obtained from all subjects. Subjects are described in detail in Supplementary Table 1. At the time of *in-vivo* period measurement, all subjects had not taken melatonin or prescribed medications. At the time of skin biopsy, some blind subjects were currently taking melatonin (see Supplementary Table 1). With the exception of one subject (Basel) for whom stable *in-vitro* measurements could not be obtained (Subject Start05), all biopsied subjects were included in our analysis.

**Circadian period determination in sighted subjects (Basel).**

One week prior to the study (baseline week), participants were requested to abstain from excessive caffeine and alcohol consumption (one caffeine-containing beverage per day at most and less than five alcoholic beverages per week). They were instructed to keep a regular sleep-wake schedule during the baseline week at home (bedtimes and wake times within ± 30 minutes of a self-selected target time between 22:00 h and 2:00 h) prior to admission to the laboratory. Compliance was checked by sleep logs and ambulatory activity measurements (wrist activity monitor, Cambridge Neurotechnology Ltd®, UK). Timing of the sleep-wake schedule during the protocol was adjusted to individual average habitual bedtimes. For each participant, habitual bedtime was calculated by centering the approximately 8-hour sleep episodes during the baseline week at their midpoint. The inpatient part of the protocol comprised two baseline 8-hour sleep episodes in darkness in the laboratory, followed by a 40-h sleep deprivation (Start study) or a 60-h multiple nap protocol with the interplay of 16 alternating sleep:wake cycles of 75:150 minutes duration (Likri study). Participants remained under constant conditions (constant dim light levels <8 lux during scheduled wakefulness, semi-recumbent posture in bed, food and liquid intake at regular intervals, no access to time cues) [1,2]. During scheduled sleep episodes a 45 degree shift to a supine posture was allowed, and the lights were turned off (0 lux). Saliva samples were collected every 20 minutes and analysed for melatonin content through a direct double-antibody radioimmunoassay (validated by gas chromatography–mass spectroscopy with an analytical least detectable dose of 0.65 pm/ml; Bühlmann Laboratories, Schönenbuch, Switzerland). The *in vivo* period length of the sighted subjects was determined via the temporal profile of melatonin content in saliva, using Cosinor^TM^ software (SEPTMR) to fit a hypothetical sinusoidal wave to the collected melatonin data. For the Start study, the temporal profile included the nights before, during, and after the constant routine; for the Likri study, the temporal profiles of the nights before and during the nap protocol were used. Data for one representative subject is shown in Figure 1A.

**Suppl. Figure. Protocol for measurement of physiological period in sighted subjects (Basel).**

Protocol for two different groups of subjects maintained in either a constant routine (START) or nap protocol (LIKRI). Crosshatched areas are scheduled sleep episodes; stippled areas reflect recumbent awake or nap periods.

**Circadian period determination in sighted subjects (Novosibirsk).**

For the current paper, subjects were recruited that participated in two chronobiological studies during which they adhered to 24-hour days with fixed sleep times in near darkness. The protocols of these studies have been described elsewhere [3,4]. Generally, the participants were required to go to bed at 23:00-23:30 for 5-14 days prior to admission to the isolation facility. Compliance was checked by wrist actimetry (Gähwiler^TM^, Zurich) and/or sleep logs. The laboratory parts included 24-h days: 9 days under <0.2 lux light ("Sleep-advance" study, #1), or 4 days under <0.1 lux red light ("ERG in DD" study, #2). The saliva for melatonin assay was collected every 0.5–3 hours on days 2, 5, and 9 during a constant routine protocol in study #1, and on days 1 and 4 in study #2. In the latter study, subjects were additionally awakened to collect night saliva samples three times during both initial and final days of measurements. (These protocols are illustrated in the Supplementary Figure below.) The melatonin content was assayed using the same kits and methods as described for Basel above. All samples from an individual were measured in a single assay. Circadian period in study #1 was obtained by linear regression across times of melatonin evening rise at each of the three days measured. Evening rise was defined as time when melatonin concentration attained 3-pg/ml threshold (by linear interpolation between two 0.5-h-apart values). In the second study, the comparison was made between two times of evening rise (on days 1 and 4) that was computed as the time of the ¼ amplitude crossing after fitting the 24 h melatonin curves with a skewed bimodal baseline cosine function [5].

**Suppl. Figure. Protocol for measurement of physiological period in sighted subjects (Novosibirsk).**

Protocol for two different groups of subjects from Novosibirsk maintained in near-complete darkness (<0.2 lux). Crosshatched areas are scheduled sleep episodes; stippled areas reflect recumbent awake periods.

**Circadian period determination in blind subjects (Guildford).**

The protocols used here to determine physiological period length of blind subjects have been reported elsewhere [6,7,8,9]. Briefly, subjects with no conscious perception of light (NPL) were studied in their own homes for 3-5 consecutive weeks. One had both eyes intact, one had one eye and 6 had no eyes (see Supplementary Table 1 All but one of the blind subjects (#S33) complained of a sleep disorder as assessed by the Pittsburgh Sleep Quality Index (PSQI; ≥ 5) [10]. No attempt was made to alter the lifestyles of the individuals during the study. For 48 hours each week, subjects collected sequential ~4-hourly urine samples during the day plus an ~8-hour overnight sample. After each collection period, the subjects measured and recorded the volume or weight of the sample and the time of the urine collection period. Urinary 6-sulphatoxymelatonin (aMT6s) concentrations were measured by radioimmunoassay using the method of Arendt et al., [11] adapted by Aldhous and Arendt [12]. Antiserum was supplied by Stockgrand Ltd., University of Surrey. Urinary data (ng/h) from each 48-hour assessment were subjected to cosinor analysis (software provided by Dr. D. S. Minors, University of Manchester, UK) to determine the acrophase, or fitted peak time, of the aMT6s rhythm. The acrophase times were subjected to regression analysis to determine the period (τ = 24 h + slope) [6].

**Measurement of fibroblast circadian period length.**

Subjects from each of the studies presented above were recontacted to participate in the present investigation by donating skin biopsies as described below. The time elapsed between measurements *in vivo* and fibroblast donation is indicated in Supplementary Table 1. During measurements of physiological period length, no subject used medications known to alter circadian phase. At the time of skin biopsy, four blind subjects were concurrently taking melatonin (2-6 mg/day in the evening), one blind participant had recently taken melatonin, and 3 blind subjects reported not taking melatonin. To ensure that this medication did not interfere with measurements *in vitro*, cells from all studies were identically cultivated for at least six weeks prior to measurement, using medium that did not contain melatonin.

Two cylindrical 2-mm diameter cutaneous biopsies were taken from the buttocks or upper arms of each subject. The time of day at which biopsies were taken varied from 10:00 to 14:00, but we have shown previously that time of biopsy does not affect measured period length. [13]. Immediately after removal, biopsies were placed in Dulbecco's Modified Eagle's Medium high glucose (DMEM)/1% Penicillin-Streptomycin solution (Sigma)/1%Glutamax (Sigma), hereafter designated as DMEMc, supplemented with 50% Foetal Bovine Serum (FBS) (Sigma), and shipped on ice to Basel, where cell isolations were conducted. Fibroblasts were isolated from biopsies by digestion of tissue for 4 hours in DMEMc supplemented with 20% Foetal Bovine Serum (FBS) (Sigma) and 87.5 ng/ml liberase (Roche). After digestion, cells were cultured in DMEMc/20%FBS. Confluent cells were infected using *Bmal1*::luciferase lentivirus as described previously [13]. Five days or more after human fibroblast infection circadian rhythms were synchronized by 100nM dexamethasone (Sigma) in DMEMc + 20% FBS [14]. DMEMc without phenol red was supplemented with 0.1 mM luciferin (Molecular Probes, USA) and light output was measured (3 measurements per biopsy; total of 6 measurements per subject) in specially built light-tight atmosphere-controlled boxes for at least 5 days. For each luciferase measurement, the period of oscillation was calculated by analyzing the period between the second and the fifth day of measurement, fitting hypothetical sine curves with period and phase as free variables using the software Lumicycle Analysis (Actimetrics, USA). The period of the sine wave with the best least-squares fit to the data was assumed to be the true period of oscillation. Values are presented as mean plus or minus standard error from four to eight measurements. A portion of the measures from a representative subject is shown in Figure 1B (the same subject whose *in-vivo* data are plotted in Figure 1A).

**REFERENCES**

1. Cajochen C, Knoblauch V, Krauchi K, Renz C, Wirz-Justice A (2001) Dynamics of frontal EEG activity, sleepiness and body temperature under high and low sleep pressure. Neuroreport 12: 2277-2281.

2. Munch M, Knoblauch V, Blatter K, Schroder C, Schnitzler C, et al. (2005) Age-related attenuation of the evening circadian arousal signal in humans. Neurobiol Aging 26: 1307-1319.

3. Danilenko KV, Cajochen C, Wirz-Justice A (2003) Is sleep per se a zeitgeber in humans? J Biol Rhythms 18: 170-178.

4. Danilenko KV, Plisov IL, Wirz-Justice A, Hebert M (2009) Human retinal light sensitivity and melatonin rhythms following four days in near darkness. Chronobiol Int 26: 93-107.

5. Van Someren EJ, Nagtegaal E (2007) Improving melatonin circadian phase estimates. Sleep Med 8: 590-601.

6. Lockley SW, Skene DJ, Arendt J, Tabandeh H, Bird AC, et al. (1997) Relationship between melatonin rhythms and visual loss in the blind. J Clin Endocrinol Metab 82: 3763-3770.

7. Skene DJ, Lockley SW, Thapan K, Arendt J (1999) Effects of light on human circadian rhythms. Reprod Nutr Dev 39: 295-304.

8. Lockley SW, Skene DJ, James K, Thapan K, Wright J, et al. (2000) Melatonin administration can entrain the free-running circadian system of blind subjects. J Endocrinol 164: R1-6.

9. Hack LM, Lockley SW, Arendt J, Skene DJ (2003) The effects of low-dose 0.5-mg melatonin on the free-running circadian rhythms of blind subjects. J Biol Rhythms 18: 420-429.

10. Buysse DJ, Reynolds CF, 3rd, Monk TH, Berman SR, Kupfer DJ (1989) The Pittsburgh Sleep Quality Index: a new instrument for psychiatric practice and research. Psychiatry Res 28: 193-213.

11. Arendt J, Bojkowski C, Franey C, Wright J, Marks V (1985) Immunoassay of 6-hydroxymelatonin sulfate in human plasma and urine: abolition of the urinary 24-hour rhythm with atenolol. J Clin Endocrinol Metab 60: 1166-1173.

12. Aldhous ME, Arendt J (1988) Radioimmunoassay for 6-sulphatoxymelatonin in urine using an iodinated tracer. Ann Clin Biochem 25 (Pt 3): 298-303.

13. Brown SA, Fleury-Olela F, Nagoshi E, Hauser C, Juge C, et al. (2005) The period length of fibroblast circadian gene expression varies widely among human individuals. PLoS Biol 3: e338.

14. Balsalobre A, Brown SA, Marcacci L, Tronche F, Kellendonk C, et al. (2000) Resetting of circadian time in peripheral tissues by glucocorticoid signaling. Science 289: 2344-2347.

**Supplementary Table 1 – Subject Information**

| Study acronym | Dura-tion | Subject  code | Sex | Age at biopsy | Year of study | Year of biopsy | Tau in vivo | Tau in vitro | Eyes intact |
| --- | --- | --- | --- | --- | --- | --- | --- | --- | --- |
| **Basel** |  |  |  |  |  |  | (cosinor) |  |  |
| Start  (Cajochen *et al.,* 2001; Munch *et al.,* 2005) | 3 days | Start 21 | m | 63 | 2002 | 2006 | 24.45 | 25.29 | 2 |
|  |  | Start 23 | f | 73 | 2002 | 2006 | 24.13 | 25.01 | 2 |
|  |  | Start 24 | m | 64 | 2002 | 2006 | 23.80 | 23.99 | 2 |
|  |  | Start 25 | m | 66 | 2003 | 2006 | 24.10 | 25.18 | 2 |
|  |  | Start 31 | f | 68 | 2003 | 2006 | 24.50 | 25.26 | 2 |
|  |  | Start 34 | f | 63 | 2003 | 2006 | 23.90 | 25.04 | 2 |
| Likri |  | Likri03 | m | 21 | 2007 | 2007 | 23.90 | 24.38 | 2 |
|  |  | Likri04 | m | 21 | 2007 | 2007 | 23.60 | 24.65 | 2 |
|  |  | Likri06 | m | 21 | 2007 | 2008 | 24.10 | 24.58 | 2 |
| **Novosibirsk** |  |  |  |  |  |  | (regres-sion) |  |  |
| Sleep-adv (Danilenko et al., 2003) | 8 days | cC | m | 29 | 2001 | 2009 | 24.08 | 24.55 | 2 |
|  |  | Dd | f | 32 | 2001 | 2009 | 23.96 | 24.75 | 2 |
|  |  | Ee | f | 31 | 2001 | 2009 | 24.23 | 24.60 | 2 |
|  |  | Gg | m | 34 | 2001 | 2009 | 24.00 | 24.63 | 2 |
|  |  | Ll | f | 42 | 2001 | 2009 | 23.98 | 24.70 | 2 |
|  |  | Mm | f | 29 | 2001 | 2009 | 23.77 | 23.90 | 2 |
| ERG in DD (Danilenko et al., 2009) | 4 days | 2b | f | 50 | 2004 | 2009 | 24.12 | 25.00 | 2 |
|  |  | 4d | f | 22 | 2005 | 2009 | 24.17 | 24.90 | 2 |
|  |  | 5e | f | 22 | 2005 | 2009 | 24.31 | 25.45 | 2 |
|  |  | 6f | f | 22 | 2005 | 2009 | 23.71 | 24.10 | 2 |
|  |  | 7g | f | 23 | 2005 | 2009 | 23.82 | 23.90 | 2 |
| **Guildford** |  |  |  |  |  |  | (regres-sion) |  |  |
|  | 3-5 weeks | S23 | m | 53 | 1995 | 2007 | 24.39 | 23.80 | 0 |
| (MT) |  | S43 | m | 47 | 1995 | 2007 | 24.13 | 24.13 | 0 |
| (MT) |  | S17 | m | 57 | 1994 | 2007 | 24.27 | 24.10 | 2 |
|  |  | S33 | m | 67 | 1995 | 2007 | 24.6 | 24.61 | 0 |
|  |  | S48 | f | 66 | 1995 | 2007 | 24.41 | 24.71 | 0 |
| (MT) |  | S45 | m | 54 | 1995 | 2007 | 24.82 | 24.17 | 0 |
| (MT) |  | S76 | m | 50 | 2000 | 2007 | 24.6 | 24.91 | 1 |
| (MT) |  | S62 | m | 49 | 1996 | 2007 | 24.78 | 25.23 | 0 |

MT – melatonin intake at time of biopsy
